# Purification of lipopeptide biosurfactant extracts obtained from a complex residual food stream using Tricine-SDS-PAGE electrophoresis

**DOI:** 10.3389/fbioe.2023.1199103

**Published:** 2023-06-06

**Authors:** A. B. Moldes, P. Álvarez-Chaver, X. Vecino, J. M. Cruz

**Affiliations:** ^1^ CINTECX (Research Center in Technologies, Energy and Industrial Processes), Chemical Engineering Department, University of Vigo, Vigo, Spain; ^2^ CACTI (Centro de Apoyo Científico y Tecnológico a la Investigación), Structural Determination and Proteomics Service, University of Vigo, Vigo, Spain

**Keywords:** corn, spontaneous fermentation, biosurfactant, purification, analysis

## Abstract

Protocols to identify lipopeptide biosurfactant extracts contained in complex residual streams are very important, as fermented agri-food matrices are potential sources of these valuable compounds. For instance, corn steep liquor (CSL), a secondary stream of the corn wet-milling industry, is composed of a mixture of microbial metabolites, produced during the corn steeping process, and other natural metabolites released from corn, that can interfere with the purification and analysis of lipopeptides. Electrophoresis could be an interesting technique for the purification and further characterization of lipopeptide biosurfactant extracts contained in secondary residual streams like CSL, but there is little existing literature about it. It is necessary to consider that lipopeptide biosurfactants, like Surfactin, usually are substances that are poorly soluble in water at acidic or neutral pH, forming micelles what can inhibit their separation by electrophoresis. In this work, two lipopeptide biosurfactant extracts obtained directly from CSL, after liquid–liquid extraction with chloroform or ethyl acetate, were purified by applying a second liquid extraction with ethanol. Following that, ethanolic biosurfactant extracts were subjected to electrophoresis under different conditions. Lipopeptides on Tricine-SDS-PAGE (polyacrylamide gels) were better visualized and identified by fluorescence using SYPRO Ruby dye than using Coomassie blue dye. The matrix-assisted laser desorption/ionization time-of-flight mass spectrometry (MALDI-TOF-MS) analysis of lipopeptide isoforms separated by electrophoresis revealed the presence of masses at 1,044, 1,058, and 1,074 m/z, concluding that Tricine-SDS-PAGE electrophoresis combined with MALDI-TOF-MS could be a useful tool for purifying and identifying lipopeptides in complex matrices.

## 1 Introduction

Corn steep liquor (CSL) is a subproduct of the corn wet-milling industry that possesses important potential as a direct source of biocompounds, including biosurfactants of microbial origin and phospholipids (Rodríguez-López et al., 2020), among others. In the last years, it has been demonstrated that a sporulated *Bacillus* named *Bacillus aneurinilyticus*, with the ability to produce biosurfactants and other antibiotic substances, grows in CSL. The biosurfactant extracts produced by this *Bacillus* strain possess similar characteristics to the lipopeptides produced by *Bacillus subtilis*, although with a different aminoacidic chain when it is fermented in commercial media (López-Prieto et al., 2021).

It is known that a specific bacilli strain can produce diverse families of lipopeptides (Ma et al., 2016; Fanaei et al., 2021; Huarachi et al., 2022). For instance, Ma et al. (Ma et al., 2016) have identified 40 variants of lipopeptides from *Bacillus megaterium;* whereas other authors (Fanaei et al., 2021; Huarachi et al., 2022) have observed that *Bacillus mojavensis*, *Bacillus amyloliquefaciens* and *B. subtilis* can produce different lipopeptides. The most abundant amino acid in the Surfactin variants detected by these authors was leucine or isoleucine, which is consistent with the composition of Surfactin also reported in other works (Baumgart et al., 1991; Hu et al., 2019). Regarding the Surfactin variants detected by Ma et al. (Ma et al., 2016), these were composed of a sequence of 4–5 leucines or isoleucines combined with glutamic acid, aspartic acid or/and valine in different positions. These authors also found that other lipopeptides like Bacillomycin D are composed of different sequences of amino acids including in this case asparagine, tyrosine, proline, glutamic acid, serine, and threonine. Concerning the lipopeptide biosurfactant Fengycin A, this is composed of a sequence containing glutamic acid, ornithine, tyrosine, threonine, alanine, leucine or isoleucine, glutamic acid and proline. This amino acid chain is like that observed in Fengycin B but with alanine replaced by valine. Regarding the lipid composition of lipopeptides, Ma et al. (Ma et al., 2016) described the presence of C12–C18 fatty acids in the biosurfactants produced by *B. megaterium* with molecular weights between 905 and 1,509 Da. It is important to mention that the culture medium used by these authors was a commercial medium composed of glucose, yeast extract, ammonium nitrate and different minerals including NaCl, MgSO_4_, KCl, KH_2_PO_4_, CuSO_4_, MnSO_4_, and FeSO_4_, consisting in a pure culture fermented by a unique *Bacillus* strain.

On the other hand, lactic acid bacteria (Hull et al., 1996) and other *Bacillus* strains that possess the capacity to produce cell-bound biosurfactants grow in CSL (López-Prieto et al., 2021). Therefore, taking into consideration the presence of lactic acid bacteria, CSL could be considered a prebiotic fermented stream with potential for obtaining different bioactive compounds in a unique extract, hence promoting a circular economy and industrial synergies with the cosmetic (Rodríguez-López et al., 2022), agrochemical (López-Prieto et al., 2020) and pharmaceutical industries (Knoth et al., 2019; Rincón-Fontán et al., 2020). Moreover, a previous work demonstrated the prebiotic character of the lipopeptide biosurfactant extract obtained from CSL as this extract promotes the growth of *Lactobacillus casei* (López-Prieto et al., 2019a) and inhibits the growth of pathogenic bacteria (López-Prieto et al., 2019b).

However, it is necessary to consider that some of these bioactive compounds like biosurfactants are complex polymeric matrices that are not easy to purify for a correct identification. Therefore, a pure culture does not exist in spontaneously fermented streams, hence many secondary metabolites can be present, apart from the natural compounds released from the kernel of corn like phospholipids, antioxidants, or fatty acids (Rodríguez-López et al., 2016; Rodríguez-López et al., 2020). Other authors have also identified the presence of *Bacillus* strains that produce biosurfactants in different sources from the food industry, although the direct extraction of biosurfactants from these substrates has not been explored (Akintayo et al., 2022).

Regarding the identification of biosurfactants coming from controlled fermentation processes, the use of liquid–liquid extraction or precipitation followed by mass spectrometry could be an option (Kügler et al., 2015). However, when biosurfactant extracts come from complex matrices, after liquid–liquid extraction with organic solvents or after precipitation, the direct use of mass spectrometry after extraction is not probably the best option as many substances can interfere with the masses of the biosurfactants contained in the extract, spectra with a huge number of signals being obtained. In these spectra, the masses of the lipopeptide biosurfactants are masked with those of other metabolites, a weak signal being observed for lipopeptides (Rodríguez-López et al., 2020; López-Prieto et al., 2022). For instance, in the biosurfactant extracts obtained from CSL, the presence of antioxidants, which can be in polymeric form given masses close to those of lipopeptides, has also been demonstrated (Rodríguez-López et al., 2016). Also, the lipopeptides included in the biosurfactant extracts obtained from CSL can form micelles, solubilizing other bioactive compounds present in this extract, forming complex molecules micelles. Altogether, these can prevent the purification and further identification of biosurfactants like lipopeptides in biosurfactant extracts mainly when they are produced in spontaneous heterogeneous microbial fermented media. Concerning this, the use of electrophoresis prior to mass spectrum analysis could be an interesting tool for the purification and identification of lipopeptides, mainly in complex matrices like corn steep liquor. However, few studies have considered electrophoresis for purifying fermented streams containing lipopeptides, probably because lipopeptides, like Surfactin, are poorly soluble in water, preventing the migration of the biosurfactant through the electrophoresis gel.

Therefore, the aim of this work was to evaluate different liquid–liquid extraction systems for the extraction of lipopeptide biosurfactants and precipitation of impurities before electrophoresis and mass spectrometry analysis, providing a methodology for detecting biosurfactants in a complex matrix like corn steep liquor. Various conditions during electrophoresis are considered in the study, including different sample carrier solutions and detection systems.

## 2 Materials and methods

### 2.1 Extraction of biosurfactant extracts from corn steep liquor

Two different biosurfactant extracts (BS1 and BS2) were obtained from CSL using chloroform (BS1) or ethyl acetate (BS2) as extractant agents, respectively. For the extraction, the protocol established in a previous work was applied (Rodríguez-López et al., 2016). The extraction with chloroform was carried out at 56°C for 60 min, using a CSL: chloroform ratio of 1: 2 (*v/v*) at 200 rpm; the extraction with ethyl acetate was carried out at 25°C for 45 min using a CSL: ethyl acetate ratio of 1: 3 (*v/v*) at 200 rpm. After finishing the extraction process, the organic and aqueous phases were separated by decantation for 24 h and then the organic phase containing the biosurfactant extract was distilled with a Büchi R-120 rotavapor (Labortechnik, Switzerland), obtaining the raw BS1 and BS2 depending on the extractant employed.

**FIGURE 1:**
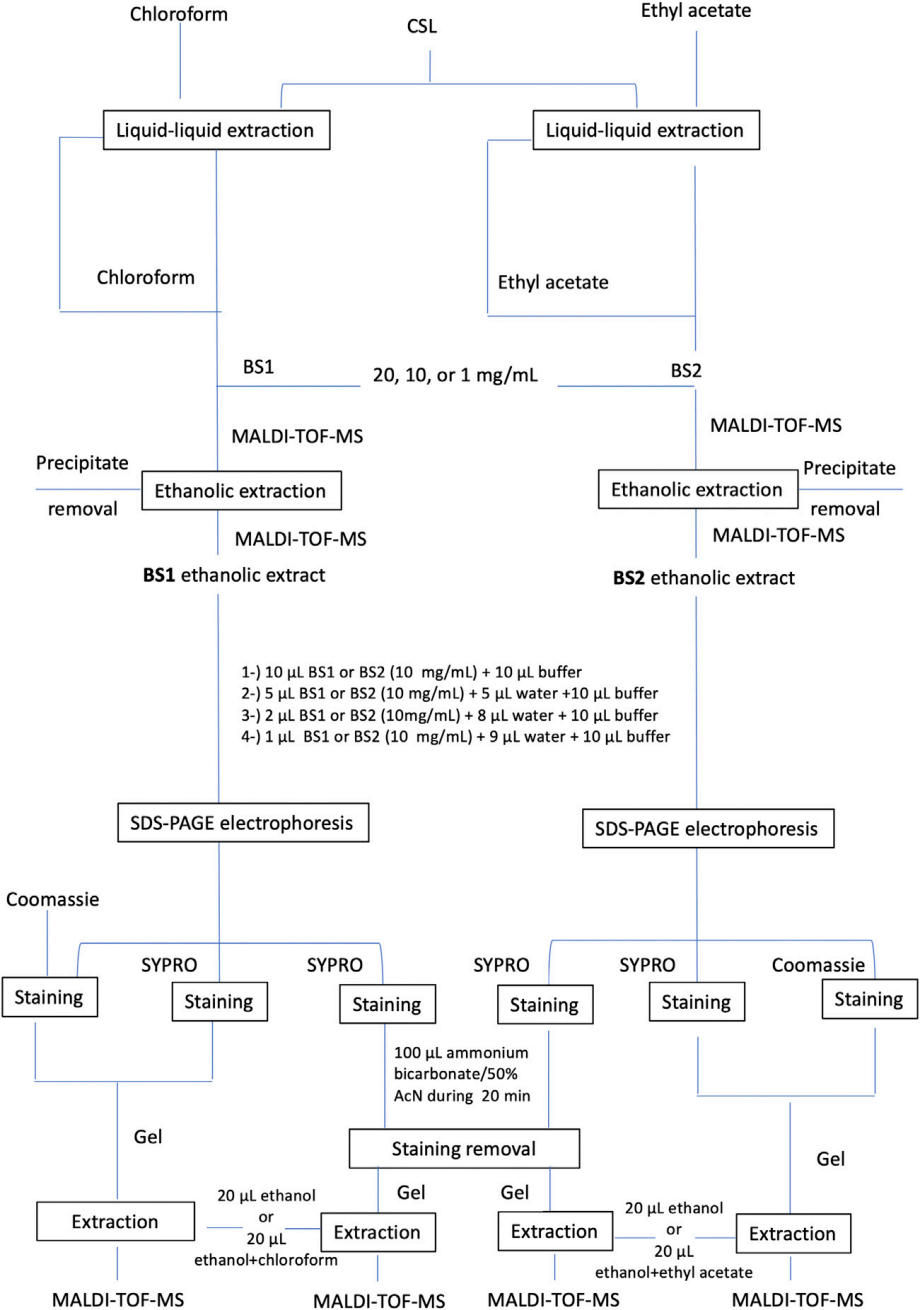
Assays established for the extraction and purification of lipopeptide biosurfactant extracts obtained from corn steep liquor.

Moreover, to achieve purer biosurfactant extracts, these were subjected to a subsequent extraction with ethanol by dissolving the biosurfactant extracts in ethanol at different concentrations (20, 10, and 1 mg of biosurfactant/mL of ethanol) and centrifuging to remove impurities. Following that, the ethanolic biosurfactant extract was recovered for analysis. Figure 1 includes the scheme followed for the purification and analysis of the biosurfactant extract obtained from the secondary residual stream under study (e.g., CSL).

### 2.2 Purification of biosurfactant extracts by Tricine-SDS-PAGE electrophoresis

After the sequential organic extractions described above, ethanolic biosurfactant extracts were subjected to Tricine sodium dodecyl sulphate polyacrylamide gel electrophoresis (Tricine-SDS-PAGE), based on a Tricine sample buffer. For that, extracts of BS1, BS2 and commercial Surfactin from Sigma Aldrich (10 mg/mL) were mixed 1: 1 with Tricine sample buffer (200 mM Tris-HCl, pH 6.8, 40% glycerol, 2% SDS, 0.04% Coomassie blue G-250% and 2% β-mercaptoethanol) and separated in polyacrylamide (40% total acrylamide and 5% crosslinker bisacrylamide) denaturing mini gels. No special cooling was used. For gels stained with SYPRO Ruby dye, stained lipopeptide bands were visualized using the UV transilluminator drawer of ChemiDoc XRS + (Bio-Rad). Two different sets of gel electrophoresis were carried out, one stained with Coomassie blue Imperial™ Protein stain (Thermo Scientific) that allows the visualization of proteins as blue bands, and the other stained with Invitrogen™ SYPRO^®^ Ruby that allows the visualization of proteins by fluorescence. A control lane was run with 10 µL of Dual Xtra Molecular Weight Standards (Bio-Rad). Lanes visualized with Coomassie blue gels were loaded with different ratios of ethanolic biosurfactant extract, water, and Tricine sample buffer. In the case of gels stained with SYPRO Ruby, lane samples contained 5 µL of BS1, BS2 or Surfactin +5 µL of water +10 µL of Tricine-SDS-PAGE buffer.

Tricine-SDS-PAGE was carried out with 16% (*v/v*) separating and 4% (*v/v*) stacking gels. The voltage was adjusted to 30 or 50 V until samples reached the running gel and then it was set at 50 or 100 V, respectively, until the end of the run. Gels were stained overnight with continuous agitation using 50 mL of Coomassie Blue or SYPRO Ruby dye. A rinse step in 10% methanol and 7% acetic acid for 1 h was included for SYPRO Ruby gels to decrease background fluorescence. Finally, gels were washed in water and images were acquired with a ChemiDoc XRS + system (Bio-Rad).

Bands were excised from the gels using a clean cutter to further analysis of lipopeptides. For gels stained with SYPRO^®^ Ruby dye, stained lipopeptide bands were visualized using the built-in UV transilluminator of ChemiDoc XRS+ (Bio-Rad).

Then, these bands were washed twice with 100 µL of ammonium bicarbonate/50% acetonitrile for 20 min to destain the samples and compare them with non-destained samples at the same electrophoretic conditions.

Finally, Surfactin was extracted from the cut band in an Eppendorf tube with 20 µL of 100% ethanol, BS1 with 20 µL of 100% ethanol or chloroform + ethanol (*v/v*) and BS2 with 20 µL of 100% ethanol or ethyl acetate + ethanol (*v/v*). Figure 1 describes the extraction and purification assays carried out for the identification of lipopeptides contained in CSL prior to matrix-assisted laser desorption/ionization time-of-flight mass spectrometry analysis (MALDI-TOF-MS).

### 2.3 Matrix-assisted laser desorption/ionization time-of-flight mass spectrometry (MALDI-TOF-MS) analysis

Samples of biosurfactant extracts (2 µL) were mixed 1: 1 with a α-cyano-4-hydroxycinnamic acid (CHCA) matrix at 3 μg/μL in ethanol: acetone (*v/v*). In order to study the effect of trifluoroacetic acid (TFA), in selected samples, 0.1% TFA was added to the CHCA matrix at a 1: 1 ratio (*v/v*). Biosurfactant extracts were then mixed with the matrix solution using a 1: 1 ratio (*v/v*). Once the samples had been prepared, they were spotted on a MTP AnchorChip™ MALDI target (Bruker Daltonik, Bremen, Germany) and allowed to air-dry. A calibration standard (Bruker Daltonik, Bremen, Germany) was used to perform external mass calibration. Mass spectra were obtained using an Autoflex III smartbeam MALDI-TOF-MS system (Bruker Daltonik, Bremen, Germany) as described in a previous study (Rincón-Fontán et al., 2017).

## 3 Results and discussion

### 3.1 Purification of lipopeptide biosurfactant extracts prior to electrophoretic isolation

Samples of raw biosurfactant, obtained after extraction of CSL with chloroform (BS1) or ethyl acetate (BS2), were subjected to a second extraction with ethanol at different concentrations of biosurfactant (20, 10, and 1 mg/mL), then centrifuged and analysed by MALDI-TOF-MS. Surfactin was included in the assay as control, also subjected to a purification step with ethanol at an intermediate concentration (10 mg/mL), similarly to the biosurfactants under evaluation. Supplementary Figure S1 shows the spectrum obtained for Surfactin, in which one can observe peaks concentrated between 1,000 and 1,120 m*/z* separated by 14 Da, consistent with CH_2_ fragments.

**FIGURE 2:**
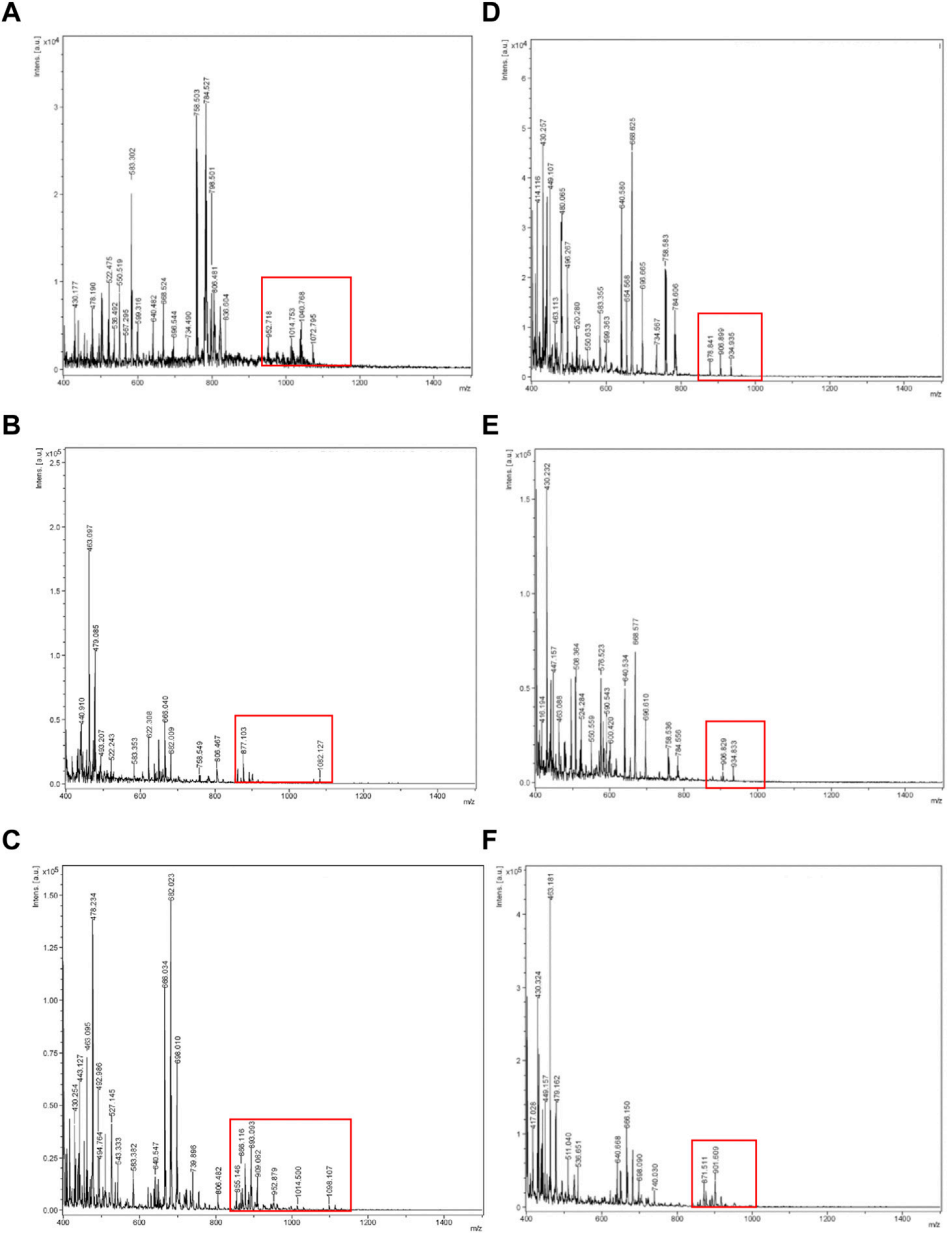
MALDI-TOF-MS spectra obtained for ethanolic BS1 and BS2 at different concentrations. **(A)** BS1 at 20 mg/mL, **(B)** BS1 at 10 mg/mL, **(C)** BS1 at 1 mg/L, **(D)** BS2 at 20 mg/mL, **(E)** BS2 at 10 mg/mL, **(F)** BS2 at 1 mg/mL.

On the other hand, Figure 2 shows the spectra obtained for compounds extracted with ethanol from the evaluated raw biosurfactants BS1 (Figures 2A–C) and BS2 (Figures 2D–F), respectively.

**FIGURE 3:**
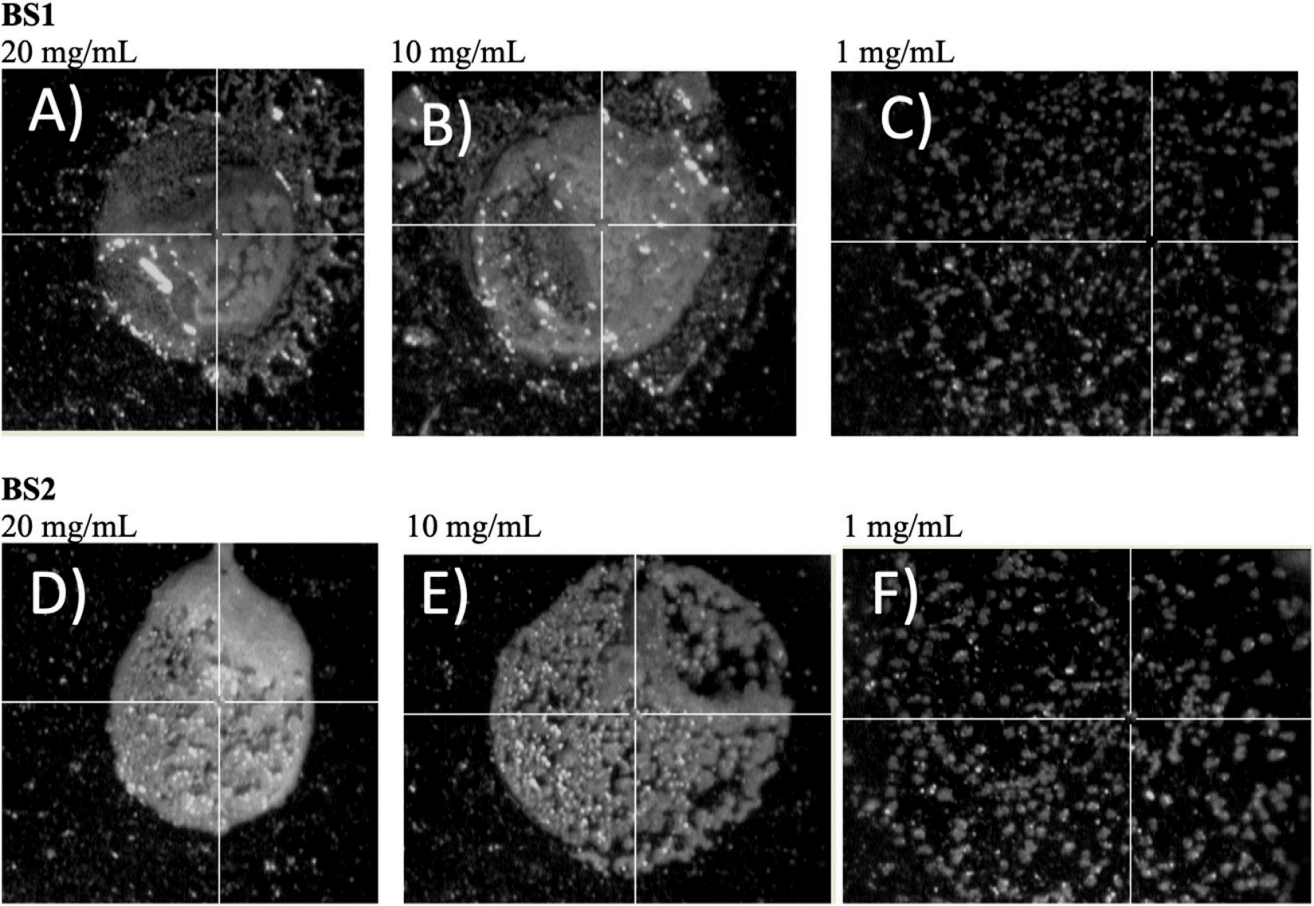
Images of the MALDI-TOF-MS target plate for the different samples of biosurfactant assayed. **(A)** BS1 at 20 mg/mL, **(B)** BS1 at 10 mg/mL, **(C)** BS1 at 1 mg/mL, **(D)** BS2 at 20 mg/mL, **(E)** BS2 at 10 mg/mL, **(F)** BS2 at 1 mg/mL.

Šebela (Šebela, 2016), in a review published about MALDI-TOF-MS analysis, recommended adding additives to the solid matrix as well as varying the analyte concentration, to get a more homogeneous crystallization on the target plate, to ensure better reproducibility and signal resolution. Based on this, in the current work different concentrations of analyte were tested in the target plate. Hence, the use of different ratios of biosurfactant extracts and ethanol, prior to MALDI-TOF-MS analysis, was proposed to obtain more refined lipopeptide biosurfactants, removing non-soluble ethanolic compounds by centrifugation, and to detect and avoid the noise produced by the formation of larger crystals when samples are highly concentrated. It can be speculated that by reducing the concentration of samples, by diluting the extract in ethanol, more homogeneous, small, and dispersed crystals can be obtained. Regarding BS1, Figures 2A–C include the spectra obtained for different samples dissolved in ethanol at different concentrations. It can be noticed that a higher concentration of BS1 (Figure 2A) produces a spectrum with more noise and less intensive signals in the range of 1,000 m*/z*. Moreover, at the lowest concentration of BS1 (1 mg/mL), more peaks around 1,000 m*/z* were observed with more intensive signals (Figure 2C), although more purification steps are needed to increase the concentration of lipopeptides to obtain more demonstrative signals for lipopeptides during MALDI-TOF-MS analysis. Similarly to BS1, it can be observed that ethanolic extracts from a lower concentration of BS2 gave more intensive signals between 800 and 1,000 m*/z* (Figure 2F). It can be speculated that higher concentrations of biosurfactants can promote the formation of concentrated crystals, reducing the ionization of samples. Figure 3 includes the camera images obtained with the MALDI-TOF-MS equipment, showing BS1 and BS2 biosurfactant extracts mixed with the CHCA matrix in the target plate, prior to MALDI-TOF-MS analysis for the different concentrations of the biosurfactant extracts in ethanol. The choice of the best matrix, solvents and sample preparation technique is a crucial step for achieving the characterization of lipopeptides in CSL, more homogeneous samples being observed in those cases working with a lower concentration of biosurfactants (1 mg/mL). Figure 3 shows the solid matrix mixed with different concentrations of biosurfactants, more heterogeneous crystallization being observed at concentrations higher than 1 mg/mL. Probably, concentrations of biosurfactant extract over 1 mg/mL promote the formation of micelles as the critical micellar concentration of the biosurfactant extracts is lower than 1 mg/mL (Rodríguez-López et al., 2020). In previous works (Rodríguez-López et al., 2016; Rodríguez-López et al., 2020), it was demonstrated that BS1 and BS2 are composed of antioxidants and other bioactive substances that can be included in the biosurfactant micelles, preventing the analysis of BS1 and BS2 at higher concentrations. Therefore, Rodrguez-López et al. (Rodríguez-López et al., 2020) demonstrated using electrospray ionization mass spectrometry/collision-induced dissociation (ESI-MS/CID) that biosurfactant extracts from CSL contain bioactive compounds corresponding with antioxidants and phospholipids consistent with the masses observed under 800 m*/z* (see Figure 2). Moreover, the signals observed at 871 and 901 m*/z* in Figure 2F were also detected by López-Prieto et al. (López-Prieto et al. 2022) in a previous work dealing with the use of the biosurfactant extract obtained from CSL as a solubilizing agent of copper oxide, but this signal was less intense than that detected in the ethanolic BS2 extract. In fact, in the general spectrum published by López-Prieto et al. (2022) the signal corresponding to lipopeptides was negligible, only observed when a zoom was applied between 850 and 1,100 m*/z*. Therefore, the above results (Figures 2, 3) indicate that extraction with chloroform is more favourable than with ethyl acetate, after that it is recommended solubilization of the biosurfactant extract in ethanol, at a concentration of 1 mg/mL, followed by centrifugation, prior to MALDI-TOF-MS analysis. Other authors (Geissler et al., 2017) have also detected differences in the extraction of lipopeptides produced by *B. amyloliquefaciens* and *Bacillus methylotrophicus* from fermented media, observing that lipopeptides ethyl acetate extracts with a lower molecular weight in comparison with chloroform and methanol (2: 1 *v/v*) give a higher concentration of Fengycin. Nevertheless, following the aim of obtaining purer lipopeptides, and studying the differences regarding the extraction process, samples of BS1 and BS2 were subjected to electrophoresis using Tricine-SDS-PAGE to separate lipopeptide biosurfactant extracts based on their molecular weight.

### 3.2 Purification of lipopeptide biosurfactants using Tricine-SDS-PAGE electrophoresis and subsequent MALDI-TOF-MS analysis

Biosurfactant extracts, BS1 and BS2, were subjected to electrophoresis analysis using Tricine-SDS-PAGE that is commonly used to separate proteins in the mass range between 1 and 100 kDa. In this method, the concentrations of acrylamide used in the gels are lower than in other electrophoretic systems, supposing less interference in the subsequent MS analysis. Moreover, the use of lower concentrations of gel facilitates electroblotting, which is particularly crucial for more hydrophobic proteins like lipopeptides similarly to Surfactin (Schägger, 2006). In comparison with the analysis of peptides and proteins, lipopeptides possess the inconvenience that they are poorly soluble in water at acidic and neutral pH, being very soluble in ethanol or methanol. Therefore, Abdel-Mawgoud et al. (2008) observed that the highest solubility of Surfactin, one of the most studied lipopeptides, was obtained at pH 8.0–8.5, while the lowest solubility observed for Surfactin was at pH 5. Moreover, lipopeptide biosurfactants can form micelles that can obstruct the migration of lipopeptides through the electrophoresis gel. Thus, it is important to find an adequate carrier that allows good solubilization of lipopeptides, promoting the migration of the biosurfactant through the gel.

**FIGURE 4:**
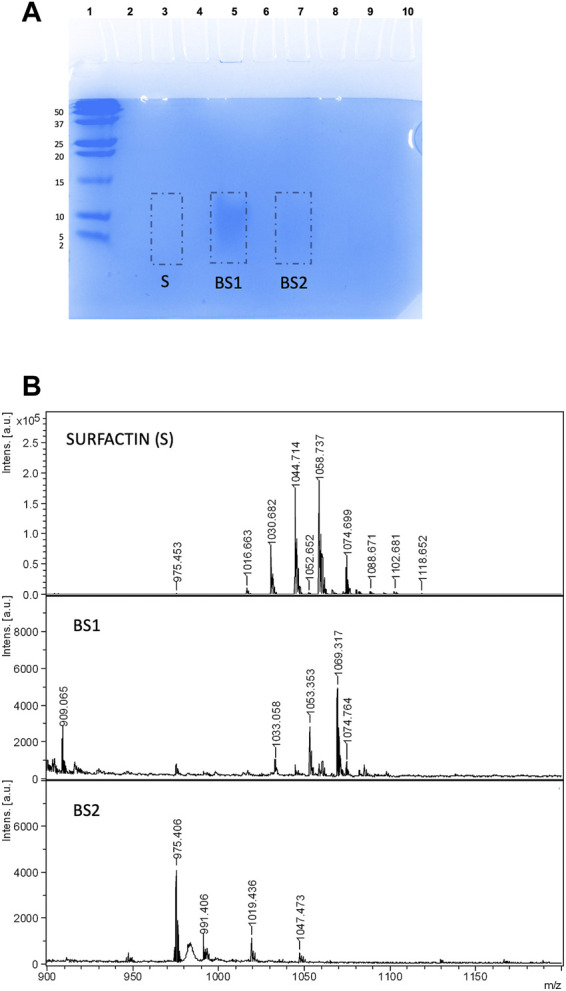
**(A)** Electrophoresis of Surfactin, BS1 and BS2 using 5 µL of ethanolic BS1 or BS2 (10 mg/mL) + 5 µL of water +10 µL of Tricine SDS-PAGE buffer stained with Coomassie blue. **(B)** MALDI-TOF-MS spectra of gel extract containing Surfactin, BS1 and BS2.

The Tricine-SDS-PAGE isolation method involves gel preparation, sample preparation, electrophoresis, protein staining or Western blotting and analysis of the generated banding pattern. In this work, two different gels were used, Coomassie blue dye and SYPRO Ruby gel stain. In a first approach, the Tricine-SDS-PAGE banding pattern of BS1 and BS2 in comparison with Surfactin stained with Coomassie blue dye gel was assayed. This method involves a single, ready-to-use reagent that does not permanently chemically modify the target of peptides/proteins; thus, peptides or protein bands can be completely destained and recovered for analysis by MS or sequencing. When samples of Surfactin or BS1 and BS2 were dissolved directly in Tricine sample buffer, following the protocol established in Figure 1, no signals were detected. This can be related to the hydrophobicity of lipopeptides in water at acidic and neutral pH that inhibits the migration of the lipopeptides through the gel; whereas diffuse bands were detected when BS1 and BS2 were dissolved in mixtures of ethanol: water: Tricine sample buffer, it being observed that BS1 gave more noticeable bands than B2 and Surfactin. Among the concentrations assayed, 5 µL of ethanolic BS1 or BS2 (10 mg/mL) + 5 µL of water +10 µL of Tricine sample buffer produced the best conditions for visualizing lipopeptides obtained from CSL when they are extracted with chloroform. Figure 4A shows the three biosurfactants (Surfactin, BS1 and BS2) after Tricine-SDS-PAGE stained with Coomassie under the conditions cited previously; a significant band is observed for BS1 (lane 5), whereas BS2 gave a negligible signal (line 7), and no signal was detected for Surfactin with this staining gel (lane 3). Thus, it can be speculated that Surfactin is more hydrophobic than BS1 and BS2. Fanaei et al. (Fanaei et al., 2021) also analysed lipopeptides produced by *B. mojavensis* using SDS-PAGE, where lipopeptides were produced in a controlled fermentation using a synthetic fermentation medium with controlled and reduced production of bioactive compounds. These authors dissolved lipopeptides in SDS-PAGE loading buffer and SDS-PAGE was carried out with 15% (w/v) separating and 5% (w/v) stacking gels, similarly to the conditions established in the current work. The voltage was adjusted to 80 V until samples reached the running gel; afterwards, it was set at 120 V (running time: 90 min). The gel was stained using 0.1% Coomassie Blue R250 in 50% methanol, 40% H_2_O and 10% acetic acid for 30 min, followed by exposure of the gel to a washing solution containing water, acetic acid and methanol (8: 1: 1 *v/v*) to remove SDS and Coomassie stain. The SDS-PAGE background obtained by Fanaei et al. (2021) for lipopeptides showed various bands corresponding to a less pure extract than those evaluated in the current work; the presence of higher molecular weight proteins was observed.

Additionally, Figure 4B shows the MALDI-TOF-MS spectra of Surfactin, BS1 and BS2 extracted in the current work from Tricine-SDS-PAGE where lipopeptides were fixed with Coomassie blue dye and extracted from the gel directly with ethanol without any additional washing step. The biosurfactants analysed were those extracted with ethanol from the pieces of gel signalized in Figure 4A. Masses in the range of 900 and 1,200 m*/z* were observed, consistent with the presence of lipopeptides. Surfactin is included in this figure for comparative purposes and to corroborate the separation of lipopeptides by electrophoresis despite the negligible signal observed in the gel. If the spectra of BS1 and BS2 included in Figure 4B are compared, it is observed that BS1 provides masses with a molecular weight in the range of Surfactin, whereas BS2 gives masses of a lower molecular weight more comparable with Kurstakin, a lower molecular weight isoform of Surfactin (Beltran-Gracia et al., 2017; Fanaei et al., 2021). Therefore, it can be speculated CSL contains different lipopeptide clusters and chloroform, the first solvent used for the extraction of BS1, produced the extraction of those clusters with higher molecular weight, whereas ethyl acetate, the first solvent used for the extraction of BS2, produced the extraction mainly of lipopeptides with lower molecular weight comparable to Kurstakin.

**FIGURE 5:**
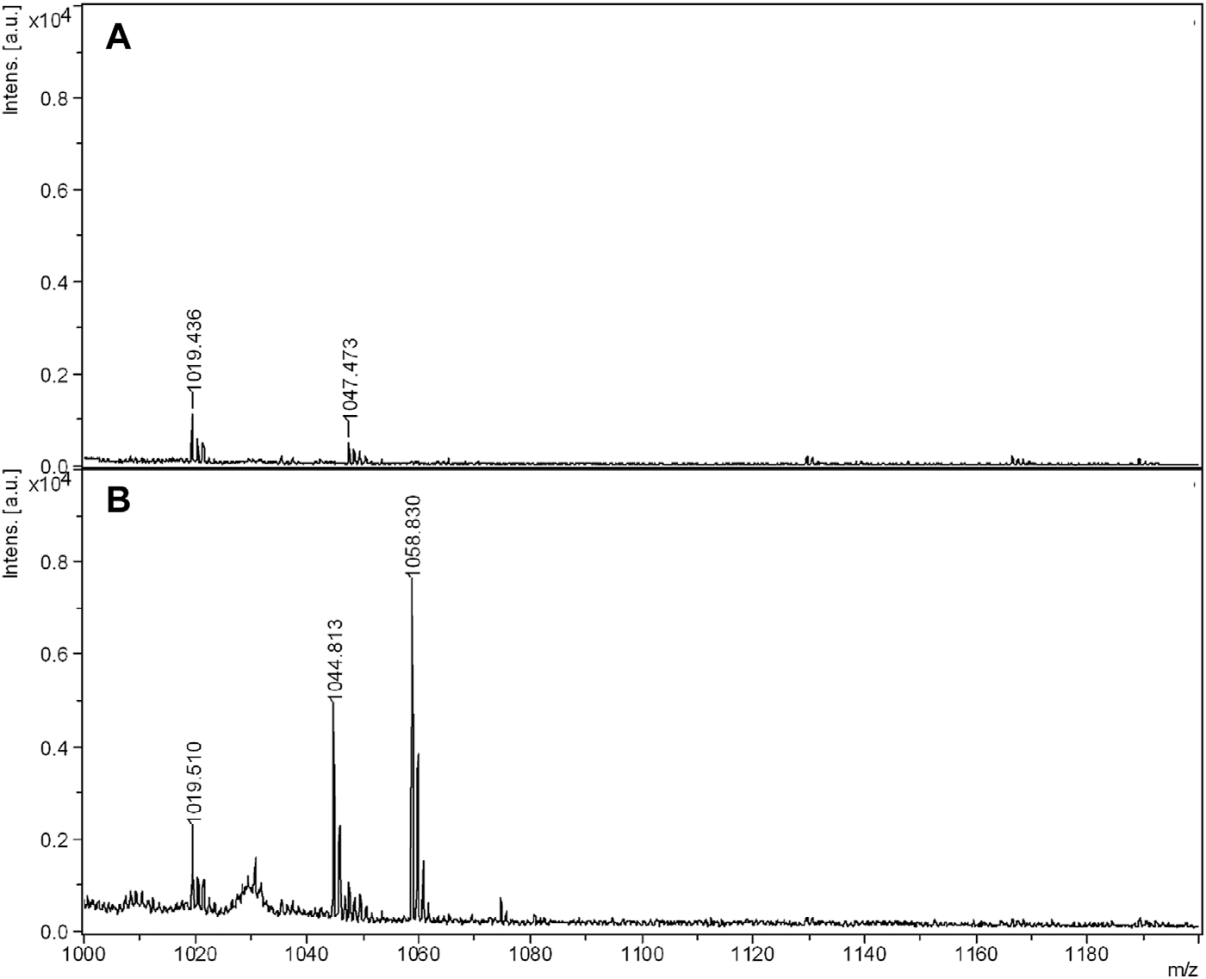
MALDI-TOF-MS spectra of non-concentrated **(A)** and concentrated **(B)** BS2.

Following that, with the aim of obtaining a better signal of lipopeptides for BS2 in the range of 1,000 m*/z*, BS2 extract eluted with ethanol from the electrophoresis gel was concentrated. Hence Figure 5 shows the MALDI-TOF-MS spectrum of BS2 after extracting the sample from Tricine-SDS-PAGE and subjecting it to a concentration process in comparison with non-concentrated BS2 extract obtained under the same conditions. When BS2 was concentrated, a better-quality MALDI-TOF-MS spectrum was obtained around 1,000 m*/z*, the existence of masses at 1,044 and 1,058 m*/z*, similarly to BS1, and a small signal at 1,074 m*/z* also being observed. Consequently, it has been observed that BS1 and BS2 possess similar lipopeptide mass signals, although BS1 has a higher concentration of lipopeptides in the range of Surfactin than BS2, whereas BS2 possesses more lipopeptides in the range of Kurstakin, which is consistent with the higher content of nitrogen for BS1 in comparison with BS2 reported in previous works (Rodríguez-López et al., 2020).

**FIGURE 6:**
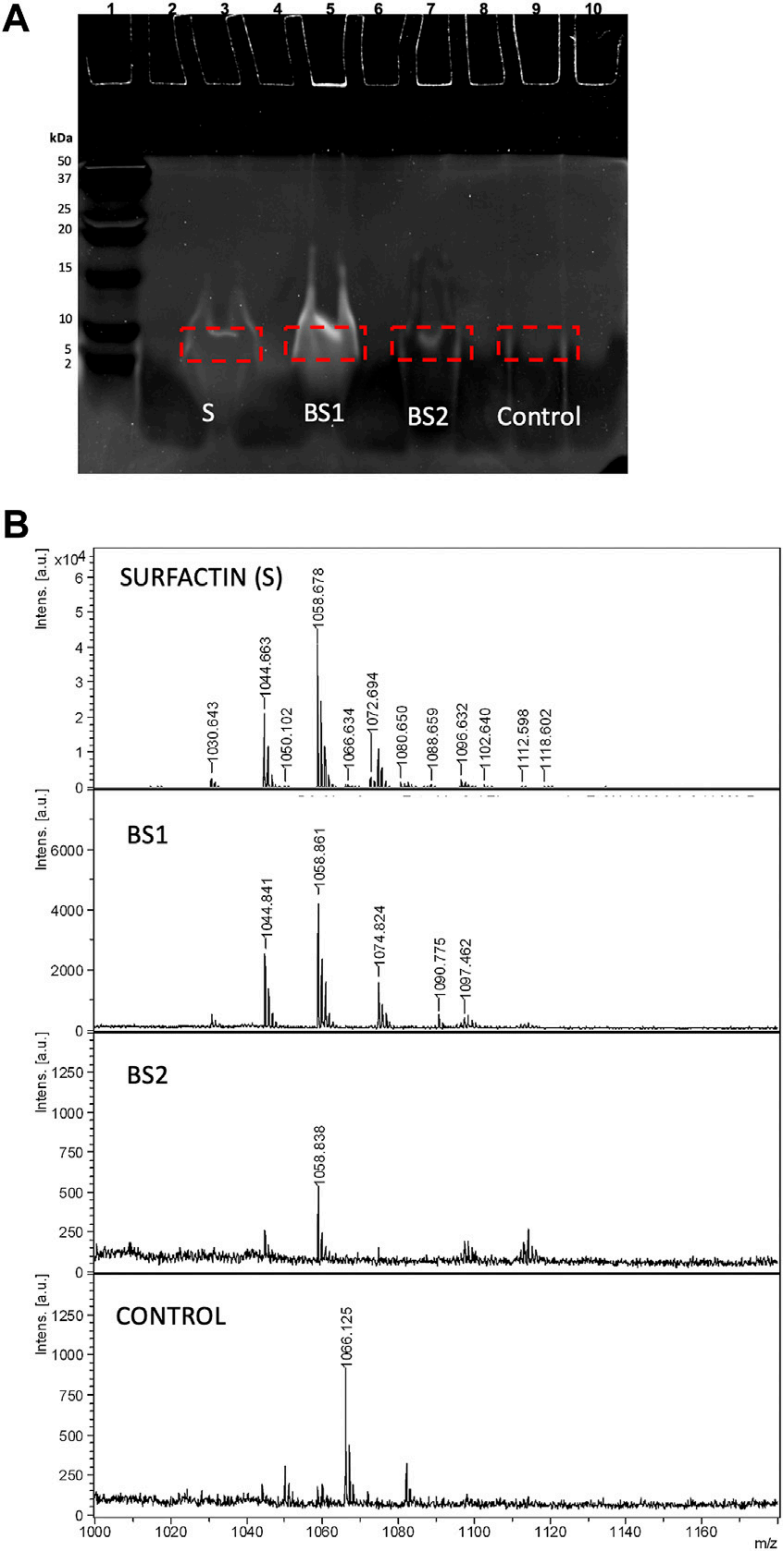
**(A)** Electrophoresis of Surfactin, BS1 and BS2 stained with SYPRO Ruby gel and visualized by fluorescence. **(B)** MALDI-TOF-MS spectra of Surfactin, BS1 and BS2 ethanolic extracts after electrophoresis as well as a control corresponding to the extract obtained from the electrophoresis gel exempt of biosurfactants.

On the other hand, to obtain a better-quality image of bands and at the same time to study the interference of dyes in the analysis of lipopeptides by MALDI-TOF-MS, Coomassie gel was replaced by SYPRO Ruby gel stain that allows the visualization of peptides and proteins in the ultraviolet range by fluorescence. It was observed that SYPRO Ruby gel stain produced more significant signals than Coomassie blue, with more convince images being observed for lipopeptides contained in BS1, BS2 and Surfactin extracts (see Figure 6A). In this case, volumes of 5 µL of biosurfactants, 5 µL of water and 10 µL of Tricine sample buffer were established. These images corroborate the similarity, regarding mass, of the lipopeptides contained in biosurfactant extracts from CSL, BS1 and BS2, corresponding with lanes 5 and 7, respectively, and Surfactin (lane 3), a more intensive band again being observed for BS1 (lane 5), similarly to the behaviour observed when using Coomassie gel if the intensities of bands ar**e** compared (Figure 4A). Moreover, Figure 6A shows the sections of gels subjected to extraction with ethanol for further MALDI-TOF-MS analysis and Figure 6B includes the MALDI-TOF-MS analysis of these samples as well as the MALDI-TOF-MS spectrum corresponding to the extraction of lane 9, exempt of biosurfactants, to notice those signals from the gel that could interfere with the masses of Surfactin and the biosurfactants under evaluation. Comparing all the spectra, again, the presence of lipopeptides with signals at 1,044, 1,058 and 1,074 m*/z* is corroborated, in the case of BS2 signals lower than 1,000 m*/z* also being observed, similarly to the MALDI-TOF-MS analysis corresponding with samples stained with Coomassie blue dye gel. Additionally, MALDI-TOF-MS spectra of BS1 were obtained for samples stained with SYPRO Ruby gel and extracted from the gel with chloroform and ethanol, signals in the range of 1,000 and 1,160 m*/z* also being observed for destained and non-destained samples, although with a higher number of signals than those observed in samples extracted from the gel with ethanol. Probably, the mixture of chloroform and ethanol extracts more substances from the gel, giving less pure extracts (see Supplementary Figures S2–S4). Regarding the stained gel, the presence of SYPRO Ruby-stained gel did not interfere with the analysis of lipopeptides by MALDI-TOF-MS, although the mass at 1,044 m*/z* was more intense in destained samples when BS1 was extracted from the gel with chloroform and ethanol (see Supplementary Figure S2). This signal at 1,044 m*/z* was observed clearly in the spectra included in Figure 6B where samples were extracted from the gel with ethanol without the destaining step. This indicates that 100% ethanol is a better extractant than the chloroform and ethanol mixture. In addition, when BS1 was extracted from the gel with ethanol and chloroform, a more intensive signal at 1,034 m*/z* was visualized (Supplementary Figure S2); this signal was also detected in the MALDI-TOF-MS spectrum of the matrix corresponding with the control (Supplementary Figure S4), thus it has not been taken into consideration. Regarding BS2, when it was extracted from the gel with a mixture of ethanol and ethyl acetate, a smaller response was observed for signals at 1,044 and 1,074 m*/z* (data not shown), a better response being shown when the extraction of the sample from the gel was carried out with 100% ethanol. Therefore, ethanol can be proposed as a unique solvent to extract biosurfactants from SDS-PAGE.

The data described above are in consonance with the spectra obtained by Fanaei et al. (2021); these authors isolated and analysed the production of Kurstakin, Fengycin and Surfactin lipopeptides produced by *B. mojavensis* using Tricine-SDS-PAGE and MALDI-TOF-MS, observing three clusters of lipopeptides in the band excised from the SDS-PAGE, corresponding to Kurstakin (600–1,000 m*/z*), Surfactin (1,000–2000 m*/z*) and Fengycin (1,400–1,600 m*/z*). Therefore, in the MALDI-TOF-MS spectrum of Surfactin, these authors also observed signals at 1,044, 1,058, 1,066, and 1,074 m*/z*, like those found in the biosurfactant extracts obtained from CSL (BS1 and BS2). Regarding the methodology used by these authors in comparison with the current work, Fanaei et al. (2021) used more reagents and time to obtain the biosurfactants from the electrophoresis gel; these lipopeptides were produced from synthetic media using controlled fermentation.

**FIGURE 7:**
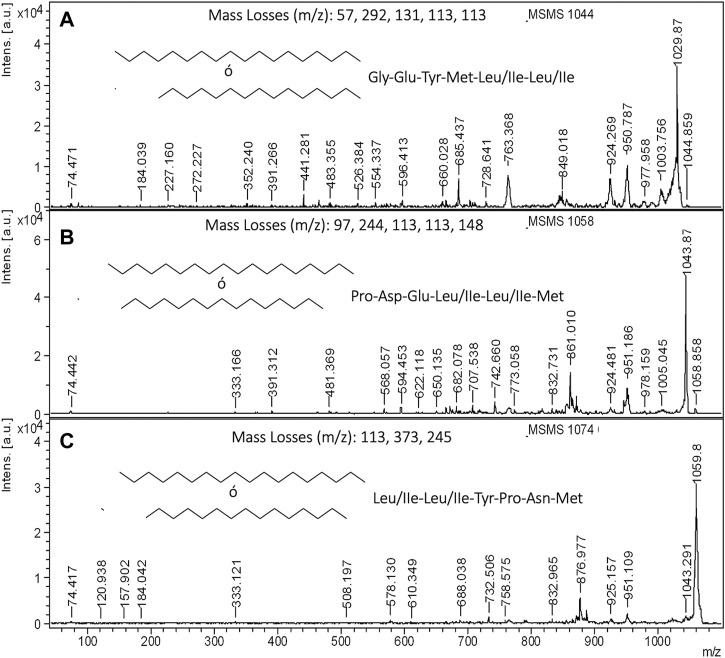
MALDI-TOF-MS/MS of lipopeptides contained in the ethanolic extract obtained from non-destained electrophoresis gel: **(A)** MS/MS of 1,044 m*/z* for BS1, **(B)** MS/MS of 1,058 m*/z* for BS1, and **(C)** MS/MS of 1,074 m*/z* for BS1.

Finally, with the aim of elucidating the aminoacidic composition of lipopeptides contained in CSL, Figure 7 shows the MALDI-TOF-MS/MS at 1,044, 1,058 and 1,074 m*/z* for BS1 extracted with chloroform. Before describing the complete aminoacidic sequence of these masses, it is interesting to point out that mass losses observed at 1,044–977, 1,058–951 and 1,074–951 m*/z* are consistent with the presence of glycine, proline and leucine or isoleucine, respectively, plus an additional mass of 10 Da in all cases (Figures 7A–C). This could be due to the chelating and amphoteric characteristics of the biosurfactants extracted from CSL demonstrated in previous works (Rodríguez-López et al., 2017; López-Prieto et al., 2022). Therefore, this difference of 10 Da could be compatible with the presence of boron that it is an element present in corn (Serbester, 2013). Consequently, the mass loss observed in the MS/MS decomposition at 1,044 m*/z* is compatible with the presence of glycine, glutamic acid, tyrosine, methionine, and leucine or isoleucine (two molecules) (Figure 7A), whereas the decomposition at 1,058 m*/z* is consistent with the presence of proline, aspartic acid, glutamic acid, leucine, or isoleucine (two molecules), and oxidate methionine (Figure 7B). In Figure 7A signal at 328 also was detected but not signalized automatically. With respect to the signal at 1,074 m*/z*, this produced losses of mass compatible with the presence of leucine or isoleucine (two molecules), tyrosine, proline, asparagine, and methionine (Figure 7C).

On the other hand when ethyl acetate was used as extractant, for obtaining BS2, it was observed a signal at 974 m*/z*, that its decomposition matches with the existence of valine, alanine (two molecules), threonine, valine, alanine, asparagine, and glycine, although in this case the losses of mass could be also consistent with other sequences containing glycine and leucine or isoleucine instead of valine and alanine (Supplementary Figure S5). Regarding the carboxylic aliphatic fatty acid chain, the residual masses observed in the spectra of Supplementary Figure S5 are compatible with the presence of fatty acids of 16–18 carbons. For instance, Gómez-Cortés et al. (2009) reported biomarkers at 164 and 292 m*/z* for linoleic acid methyl ester, that are consistent with the fragments (164 and 291 m*/z*) observed also in the decomposition of 974 m*/z*. These authors also reported that linoleic methyl esters show precursors at 320–348 m*/z* that are compatible with the biomarker observed at 333 m*/z* (Figures 7B,C), whereas the biomarkers at 184 and 227 m*/z* (Figure 7A) are compatible with methyl palmitate (Christie and Han, 2010). In most of these MALDI-TOF-MS spectra of methyl fatty acid esters, a repetitive signal is observed at 74 m*/z*, that is also observed in the MALDI-TOF-MS spectra of Figure 7. In Figure 7A, the biomarker at 74 m*/z* could also be attributed to the immonium ion mass of threonine (amino acid detected in the peptide sequence); however, the presence of the biomarkers at 184 and 227 m*/z* reinforces the presence of methyl palmitate. Concerning the carboxylic aliphatic fatty acid composition of biosurfactants, the MALDI-TOF-MS spectra included in Figure 7 are in consonance with the data reported by Rodríguez-López et al. (2020), who observed that the lipopeptide biosurfactants contained in CSL are composed of C16 and C18 methyl fatty acid esters. In the literature, it has been reported that microorganisms can produce methylated lipopeptides to the fermentation medium. Therefore, Xiang-Yang et al. (Liu et al., 2009) described the production of Surfactin mono-methyl ester by *B. subtilis* and Li et al. (2010) reported the production of lipopeptide methyl esters by *Bacillus licheniformis*.

On the other hand, in a previous work (Rodríguez-López et al., 2020), hydrolysis of the biosurfactant extract obtained from CSL was carried out after liquid–liquid extraction with chloroform and the peptide fragments were analysed by ESI-MS/MS/CID; the presence of cysteine, glutamic acid, glutamine, aspartic acid, asparagine, glycine, alanine, arginine, proline and leucine or isoleucine was reported, which is consistent with the characteristics of most amino acids obtained in the current work. Also, Yang et al. (Yang et al., 2015) carried out the identification of lipopeptide clusters (Iturin, Surfactin and Fengycin) and found major signals at 1,043 m*/z* for Iturin and at 1,030, 1,044, 1,058 and 1,072 m*/z* for Surfactin, whereas Fengycin produced major signals at 1,463, 1,477 and 1,505 m*/z*. The major amino acid in the Surfactin cluster detected by these authors was leucine whereas in Iturin and Fengycin the main amino acids were asparagine and glutamic acid, respectively. Other authors (Horn et al., 2013) have reported the presence of residues of threonine and glutamate as well as tyrosine and isoleucine in a Fengycin biosurfactant extract. In addition, Athukorala et al. (Athukorala et al., 2009) evaluated 21 *Bacillus* species including *B. subtilis*, *B. amyloliquefaciens*, *Bacillus mycoides* and *Bacillus thuringiensis*, showing the presence of specific genes related to the production of various types of biosurfactants (Bacillomycin D, Iturin A, Surfactin, Mycosubtilin, Fengycin and Zwittermicin A). Moreover, three species (*B. subtilis*, *B. amyloliquefaciens* and *B. mycoides*) were positive for Bacillomycin D, three species (*B. subtilis*, *B. amyloliquefaciens* and *B. mycoides*) were positive for Fengycin and three species (*B. mycoides*, *B. thuringiensis* and *B. amyloliquefaciens*) were positive for Zwittermicin A. These authors grew these strains in controlled fermentations at 32°C for 16–18 h to analyse DNA, which was extracted from cells with cetyltrimethylammonium bromide (CTAB), whereas for the analysis of biosurfactants strains were grown on solid synthetic medium containing glucose, L-glutamic acid, and several minerals. Following that, cells from plates were suspended in acetonitrile with 0.1% TFA for 1–2 min and then pelleted by centrifugation and the cell-free supernatant was subjected to MALDI-TOF-MS analysis using a dihydroxy benzoic acid matrix solution (DHB) in 1 mL of a solution containing 70% acetonitrile and 0.1% TFA. The mass range of Fengycin detected by Athukorala et al. (Athukorala et al., 2009) was 1,047–1,543 m*/z* depending on the protonation of masses or formation of sodium and potassium adducts; whereas Surfactin, Iturin and Bacillomycin were in the ranges 1,008–1,074, 1,070–1,150 and 1,030–1,111 m*/z*, respectively, including the presence of specific masses at 1,044, 1,074, and 1,058 m*/z*, similarly to those detected in the biosurfactant extract obtained from CSL. As can be observed, different *Bacillus* species possess the capacity to produce similar biosurfactant extracts with a similar weight, although the aminoacidic chain can vary depending on the fermentation media, operational conditions, and *Bacillus* species.

Recently, other authors (Huarachi et al., 2022) have analysed biosurfactants from *B. amyloliquefaciens* and *B. subtilis* produced in controlled fermentation using Luria Bertani (LB) broth for 6 days at 37°C. Regarding the downstream process, in this case, biosurfactants from the fermentation broth were precipitated using HCl at pH 2 and extracted with methanol that was evaporated in a following step; the extract obtained was dissolved in distilled water and the pH adjusted to 8 using 0.5 M NaOH. A different group of signals were detected using MALDI-TOF-MS. Signals compatible with the presence of Kurstakins were observed at 901, 915, 929, 943, 957, and 971 m*/z*; whereas signals consistent with Bacillomycin were detected at 1,053, 1,067, 1,068, 1,081, 1,080 and 1,097 m*/z*; for Surfactin signals were observed at 988, 1,002, 1,016, 1,030, 1,036, 1,044, 1,058, 1,074, 1,088 and 1,102 m*/z*; whereas Fengycin gave signals between 1,515 and 1,561 m*/z* depending on the adduct formation. These data are also coherent with those obtained by Athukorala et al. (Athukorala et al., 2009) and those obtained in the current work, corroborating that microorganisms, even using pure culture, do not produce a unique biosurfactant but a mixture of biosurfactants with different molecular weights and with variations in the sequence of amino acids, even between the same family of biosurfactants.

## 4 Conclusion

Based on the results described above, it can be established that the identification of lipopeptide biosurfactants in agri-food streams can be better accomplished if they are extracted with chloroform rather than ethyl acetate, followed by distillation of the organic phase and subsequent precipitation of non-soluble ethanolic compounds with ethanol. Following that, 5 µL of ethanolic extract should be mixed with 5 µL of water and 10 µL of Tricine-SDS-PAGE buffer, prior to electrophoresis. The choice of the mobile phase is a critical step as lipopeptides are more hydrophobic than proteins and peptides, which can reduce their migration through the gel. After that, samples must be stained to achieve the visualization of the lipopeptides in the gel; in this case it was observed that SYPRO Ruby gel stain gave more relevant signals (observed by fluorescence in the ultraviolet range) than Coomassie blue dye. Finally, lipopeptides can be extracted from the gel directly with ethanol, without a destaining step, and analysed by MALDI-TOF-MS/MS using a CHCA matrix. Therefore, this work can help to purify and characterized lipopeptides in complex matrices using SDS-PAGE electrophoresis, providing data on which is the more correct extractant and mobile phase.

## Data Availability

The raw data supporting the conclusion of this article will be made available by the authors, without undue reservation.

## Supplementary material

Supplementary Figure S1 MALDI-TOF-MS of commercial Surfactin between 900 and 1140 m/z dissolved in ethanol before electrophoresis assay.

Supplementary Figure S2 MALDI-TOF-MS of BS1 extracted from electrophoresis gel with chloroform and ethanol, no destained **(A)** and destained **(B)**.

Supplementary Figure S3 MALDI-TOF-MS of Surfactin extracted from electrophoresis gel with ethanol, no destained **(A)** and destained **(B)**.

Supplementary Figure S4 MALDI-TOF-MS of electrophoresis gel, extent of biosurfactant, extracted with ethanol, no destained **(A)** and destained **(B)**.

Supplementary Figure S5 MALDI-TOF-MS/MS of lipopeptide contained in BS2 at 974 m/z extracted with ethanol from non-distained electrophoresis gel.

## References

[B1] Abdel-Mawgoud A. M. Aboulwafa M. M. Hassouna N. A. H. (2008). Characterization of surfactin produced by Bacillus subtilis isolate BS5. Appl. Biochem. Biotechnol. 150, 289–303. 10.1007/s12010-008-8153-z 18437297

[B2] Akintayo S. O. Treinen C. Vahidinasab M. Pfannstiel J. Bertsche U. Fadahunsi I. (2022). Exploration of surfactin production by newly isolated Bacillus and Lysinibacillus strains from food-related sources. Lett. Appl. Microbiol. 75, 378–387. 10.1111/lam.13731 35486075

[B3] Athukorala S. N. P. Fernando W. G. D. Rashid K. Y. (2009). Identification of antifungal antibiotics of Bacillus species isolated from different microhabitats using polymerase chain reaction and MALDI-TOF mass spectrometry. Can. J. Microbiol. 55, 1021–1032. 10.1139/W09-067 19898544

[B4] Baumgart F. Kluge B. Ullrich C. Vater J. Ziessow D. (1991). Identification of amino acid substitutions in the lipopeptide surfactin using 2D NMR spectroscopy. Biochem. Biophys. Res. Commun. 177, 998–1005. 10.1016/0006-291X(91)90637-M 1905540

[B5] Beltran-Gracia E. Macedo-Raygoza G. Villafaña-Rojas J. Martinez-Rodriguez A. Chavez-Castrillon Y. Y. Espinosa-Escalante F. M. (2017). Production of lipopeptides by fermentation processes: Endophytic bacteria, fermentation strategies and easy methods for bacterial selection,” in Ferment. Process (Oily Press). 10.5772/64236

[B6] Christie W. W. Han X. (2010). Lipid analysis: Isolation, separation, identification and lipidomic analysis. Oily Press.

[B7] Fanaei M. Jurcic K. Emtiazi G. (2021). Detection of simultaneous production of kurstakin, fengycin and surfactin lipopeptides in Bacillus mojavensis using a novel gel-based method and MALDI-TOF spectrometry. World J. Microbiol. Biotechnol. 37, 97–11. 10.1007/s11274-021-03064-9 33969441

[B8] Geissler M. Oellig C. Moss K. Schwack W. Henkel M. Hausmann R. (2017). High-performance thin-layer chromatography (HPTLC) for the simultaneous quantification of the cyclic lipopeptides Surfactin, Iturin A and Fengycin in culture samples of Bacillus species. J. Chromatogr. B Anal. Technol. Biomed. Life Sci. 1045, 214–224. 10.1016/j.jchromb.2016.11.013 28153674

[B9] Gómez-Cortés P. Tyburczy C. Brenna J. T. Juárez M. de la Fuente M. A. (2009). Characterization of cis-9 trans-11 trans-15 C18:3 in milk fat by GC and covalent adduct chemical ionization tandem MS. J. Lipid Res. 50, 2412–2420. 10.1194/jlr.M800662-JLR200 19542528 PMC2781313

[B10] Horn J. N. Cravens A. Grossfield A. (2013). Interactions between fengycin and model bilayers quantified by coarse-grained molecular dynamics. Biophys. J. 105, 1612–1623. 10.1016/j.bpj.2013.08.034 24094402 PMC3822635

[B11] Hu F. Liu Y. Li S. (2019). Rational strain improvement for surfactin production: Enhancing the yield and generating novel structures. Microb. Cell. Fact. 18, 42–13. 10.1186/s12934-019-1089-x 30819187 PMC6394072

[B12] Huarachi S. F. Petroselli G. Erra-Balsells R. Audisio M. C. (2022). Antibacterial activity against enterovirulent *Escherichia coli* strains from Bacillus amyloliquefaciens B31 and Bacillus subtilis subsp. subtilis C4: MALDI-TOF MS profiling and MALDI TOF/TOF MS structural analysis on lipopeptides mixtures. J. Mass Spectrom. 57, 48966–e4910. 10.1002/jms.4896 36426779

[B13] Hull S. R. Yang B. Y. Venzke D. Kulhavy K. Montgomery R. (1996). Composition of corn steep water during steeping. J. Agric. Food Chem. 44, 1857–1863. 10.1021/jf950353v

[B14] Knoth D. Rincón-Fontán M. Stahr P. L. Pelikh O. Eckert R. W. Dietrich H. (2019). Evaluation of a biosurfactant extract obtained from corn for dermal application. Int. J. Pharm. 564, 225–236. 10.1016/j.ijpharm.2019.04.048 31004716

[B15] Kügler J. H. Le Roes-Hill M. Syldatk C. Hausmann R. (2015). Surfactants tailored by the class Actinobacteria. Front. Microbiol. 6, 212. 10.3389/fmicb.2015.00212 25852670 PMC4365757

[B16] Li Y. Yang S. Mu B. (2010). Structural characterization of lipopeptide methyl esters produced by Bacillus licheniformis HSN 221. Chem. Biodivers. 7, 2065–2075. 10.1002/cbdv.200900155 20730970

[B17] Liu X. Y. Yang S. Z. Mu B. Z. (2009). Production and characterization of a C15-surfactin-O-methyl ester by a lipopeptide producing strain Bacillus subtilis HSO121. Process Biochem. 44, 1144–1151. 10.1016/j.procbio.2009.06.014

[B18] López-Prieto A. Moldes A. B. Cruz J. M. Pérez Cid B. (2020). Towards more ecofriendly pesticides: Use of biosurfactants obtained from the corn milling industry as solubilizing agent of copper oxychloride. J. Surfactants Deterg. 23, 1055–1066. 10.1002/jsde.12463

[B19] López-Prieto A. Moldes A. B. Cruz J. M. Pérez-Cid B. (2022). Solubilization of cuprous oxide in water using biosurfactant extracts from corn steep liquor: A comparative study. Sci. Rep. 12, 2695–2712. 10.1038/s41598-022-06386-2 35177682 PMC8854742

[B20] López-Prieto A. Rodríguez-López L. Rincón-Fontán M. Cruz J. M. Moldes A. B. (2021). Characterization of extracellular and cell bound biosurfactants produced by Aneurinibacillus aneurinilyticus isolated from commercial corn steep liquor. Microbiol. Res. 242, 126614. 10.1016/j.micres.2020.126614 33045681

[B21] López-Prieto A. Rodríguez-López L. Rincón-Fontán M. Moldes A. B. Cruz J. M. (2019a). Effect of biosurfactant extract obtained from the corn-milling industry on probiotic bacteria in drinkable yogurt. J. Sci. Food Agric. 99, 824–830. 10.1002/jsfa.9251 30003538

[B22] López-Prieto A. Vecino X. Rodríguez-López L. Moldes A. B. Cruz J. M. (2019b). A multifunctional biosurfactant extract obtained from corn steep water as bactericide for agrifood industry. Foods 8, 410. 10.3390/foods8090410 31547439 PMC6769998

[B23] Ma Y. Kong Q. Qin C. Chen Y. Chen Y. Lv R. (2016). Identification of lipopeptides in Bacillus megaterium by two-step ultrafiltration and LC–ESI–MS/MS. Amb. Express 6, 79. 10.1186/s13568-016-0252-6 27639854 PMC5026979

[B24] Rincón-Fontán M. Rodríguez-López L. Vecino X. Cruz J. M. Moldes A. B. (2017). Influence of micelle formation on the adsorption capacity of a biosurfactant extracted from corn on dyed hair. RSC Adv. 7, 16444–16452. 10.1039/c7ra01351e

[B25] Rincón-Fontán M. Rodríguez-López L. Vecino X. Cruz J. M. Moldes A. B. (2020). Potential application of a multifunctional biosurfactant extract obtained from corn as stabilizing agent of vitamin C in cosmetic formulations. Sustain. Chem. Pharm. 16, 100248. 10.1016/j.scp.2020.100248

[B26] Rodríguez-López L. Rincón-Fontán M. Vecino X. Cruz J. M. Moldes A. B. (2020). Extraction, separation and characterization of lipopeptides and phospholipids from corn steep water. Sep. Purif. Technol. 248, 117076. 10.1016/j.seppur.2020.117076

[B27] Rodríguez-López L. Rincón-Fontán M. Vecino X. Cruz J. M. Moldes A. B. (2022). Study of biosurfactant extract from corn steep water as a potential ingredient in antiacne formulations. J. Dermatol. Treat. 33, 393–400. 10.1080/09546634.2020.1757016 32297562

[B28] Rodríguez-López L. Rincón-Fontán M. Vecino X. Cruz J. M. Moldes A. (2017). Ionic behavior assessment of surface-active compounds from corn steep liquor by exchange resins. J. Surfactants Deterg. 20, 207–217. 10.1007/s11743-016-1897-5

[B29] Rodríguez-López L. Vecino X. Barbosa-Pereira L. Moldes A. B. Cruz J. M. (2016). A multifunctional extract from corn steep liquor: Antioxidant and surfactant activities. Food Funct. 7, 3724–3732. 10.1039/c6fo00979d 27492045

[B30] Schägger H. (2006). Tricine-SDS-PAGE. Nat. Protoc. 1, 16–22. 10.1038/nprot.2006.4 17406207

[B31] Šebela M. (2016). Solid mixed matrices and their advantages in matrix-assisted laser desorption/ionisation time-of-flight mass spectrometry. Spectrosc. Eur. 28, 10–14.

[B32] Serbester U. (2013). Determination of boron level in feeds used in cattle nutrition in regions of central anatolia and mediterranean of Turkey. Kahramanmaraş Sütçü İmam Üniversitesi Doğa Bilim. Derg. 16, 25–27.

[B33] Yang H. Li X. Li X. Yu H. Shen Z. (2015). Identification of lipopeptide isoforms by MALDI-TOF-MS/MS based on the simultaneous purification of iturin, fengycin, and surfactin by RP-HPLC. Anal. Bioanal. Chem. 407, 2529–2542. 10.1007/s00216-015-8486-8 25662934

