# Supplementary figures and images for: Purification of lipopeptide biosurfactant extracts obtained from a complex residual food stream using Tricine-SDS-PAGE electrophoresis
Source: Front Bioeng Biotechnol. 2023 Jun 6;11:1199103. doi: 10.3389/fbioe.2023.1199103 (PMC10280073; doi:10.3389/fbioe.2023.1199103)

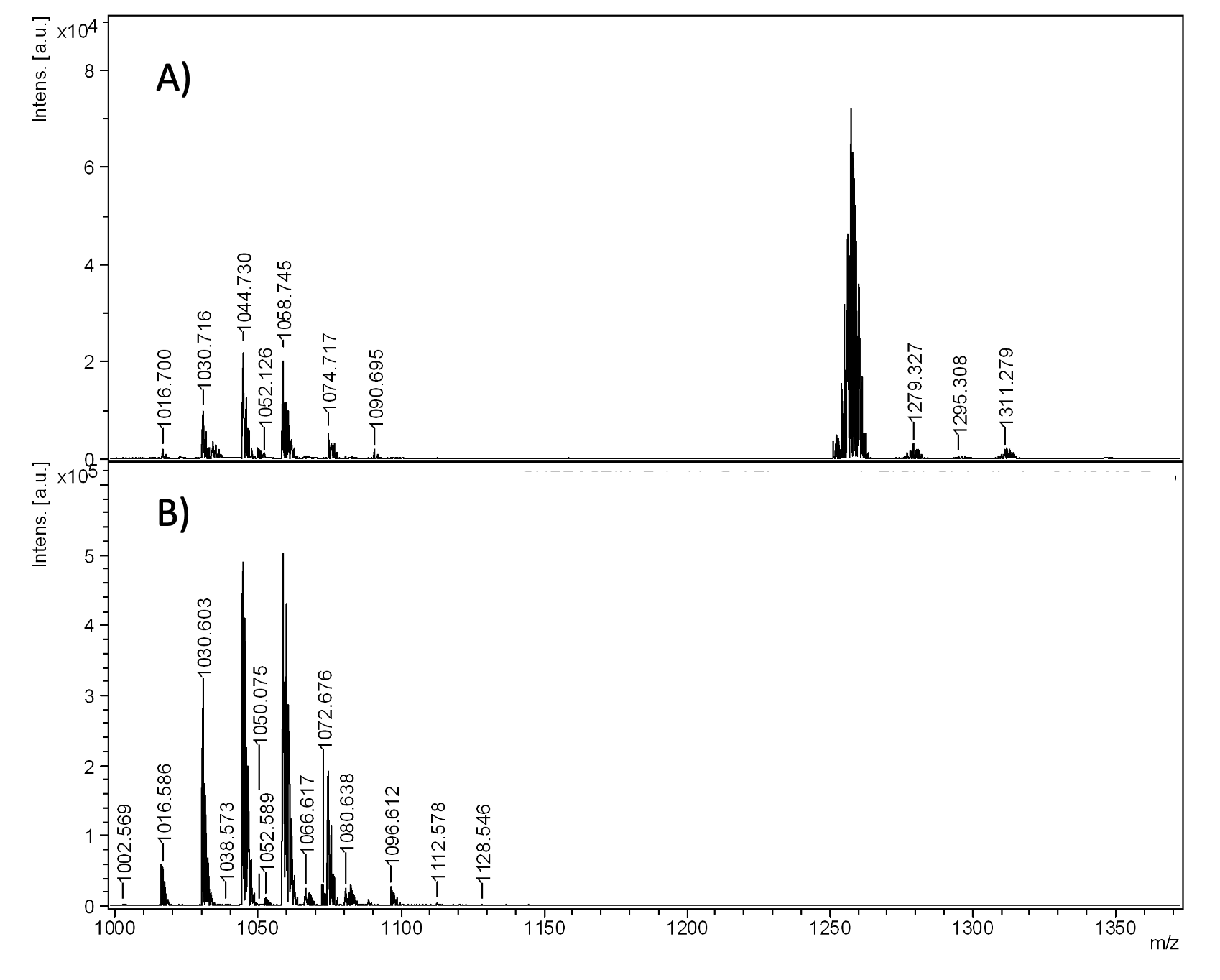

Supplement: Supplementary file 1 [file Image3.TIFF]

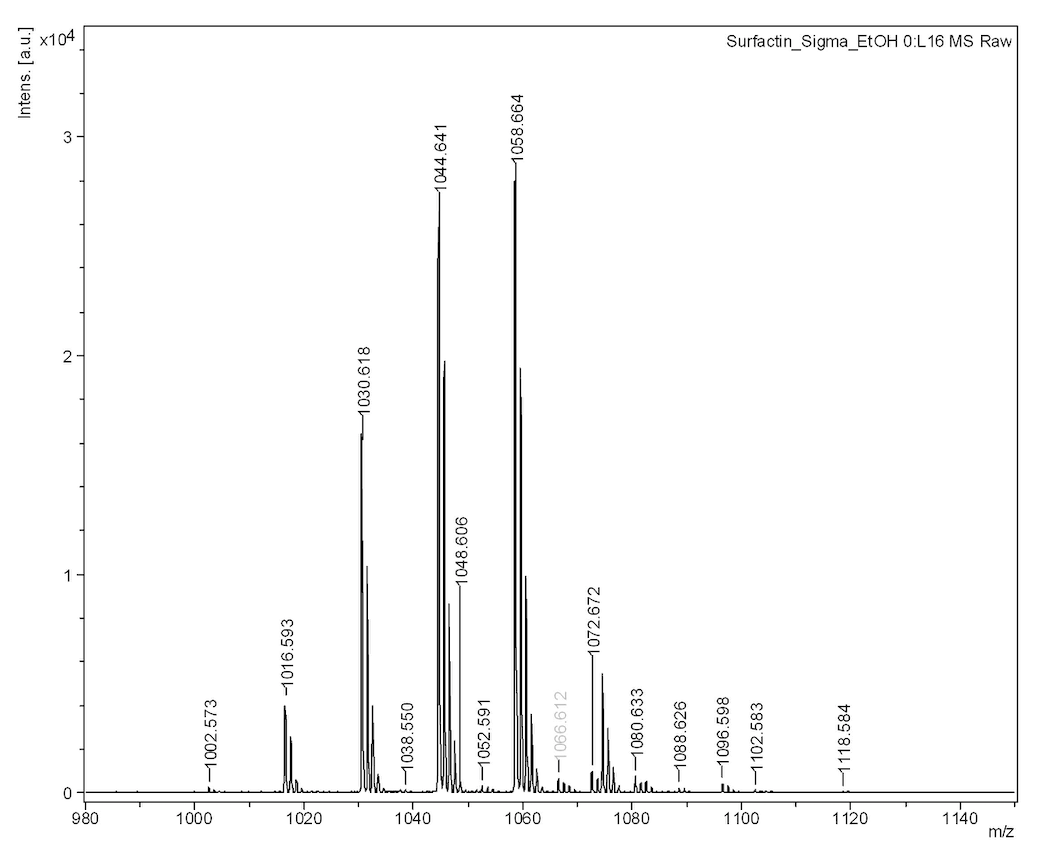

Supplement: Supplementary file 2 [file Image1.tiff]

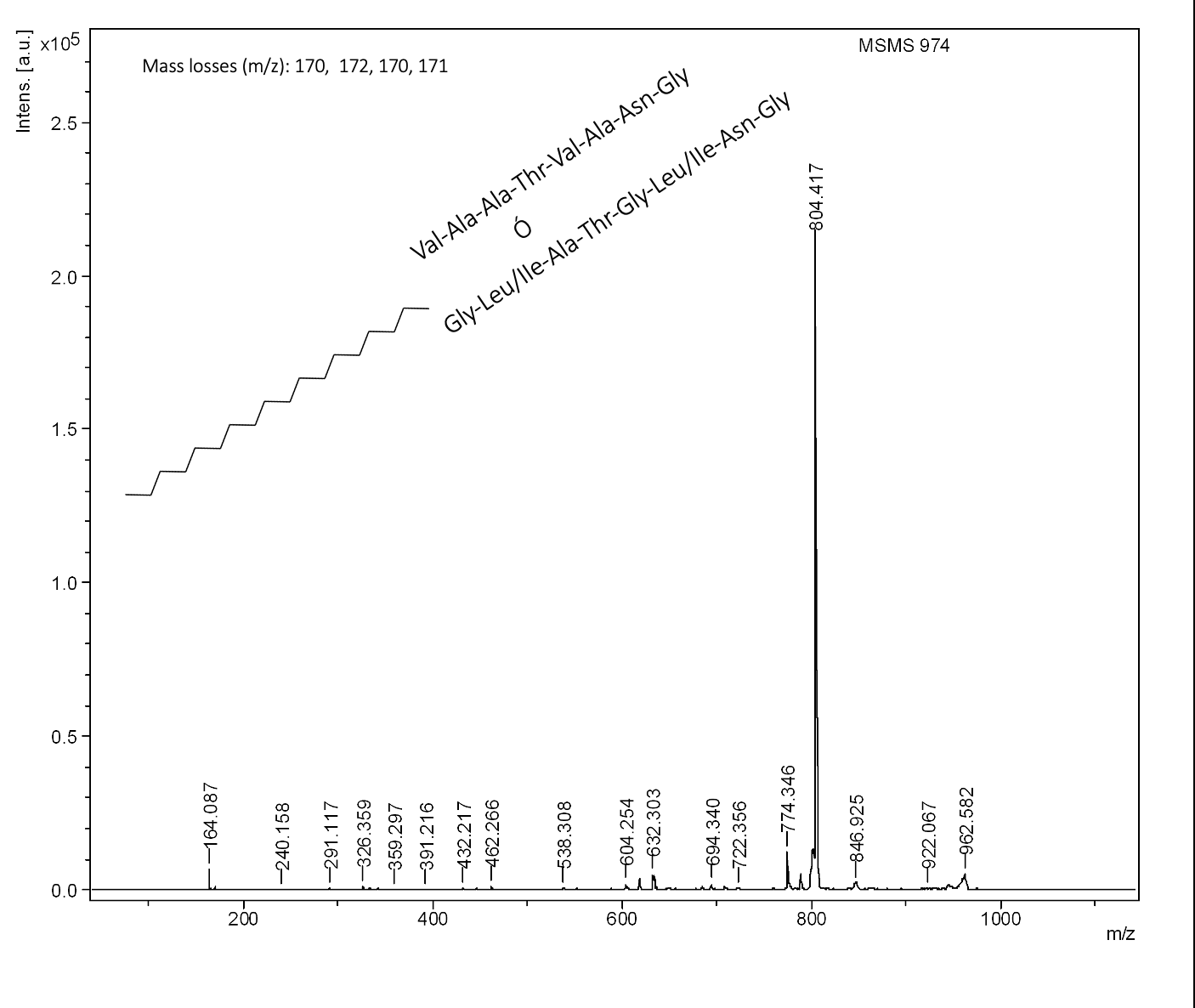

Supplement: Supplementary file 3 [file Image5.TIFF]

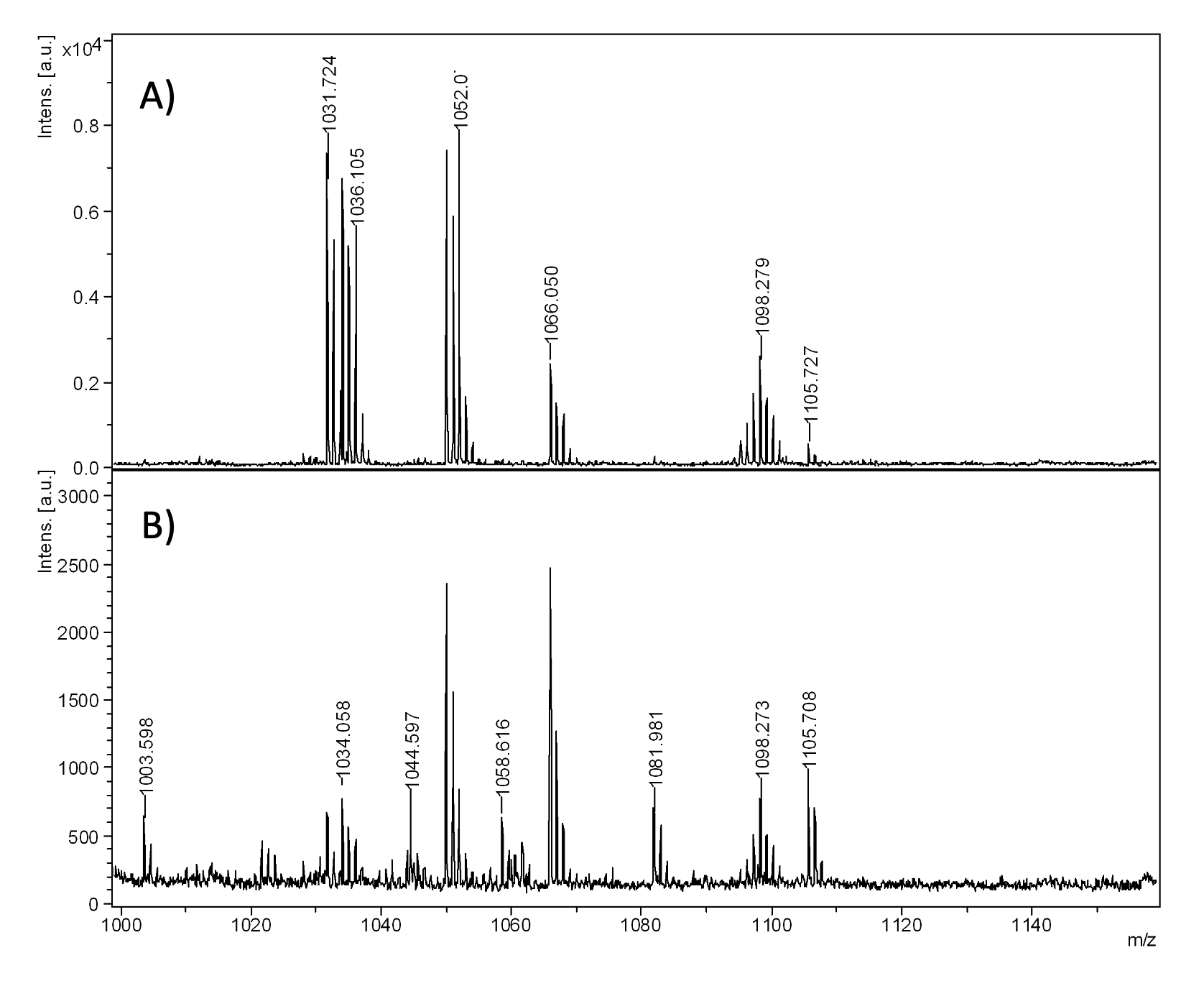

Supplement: Supplementary file 4 [file Image2.TIFF]

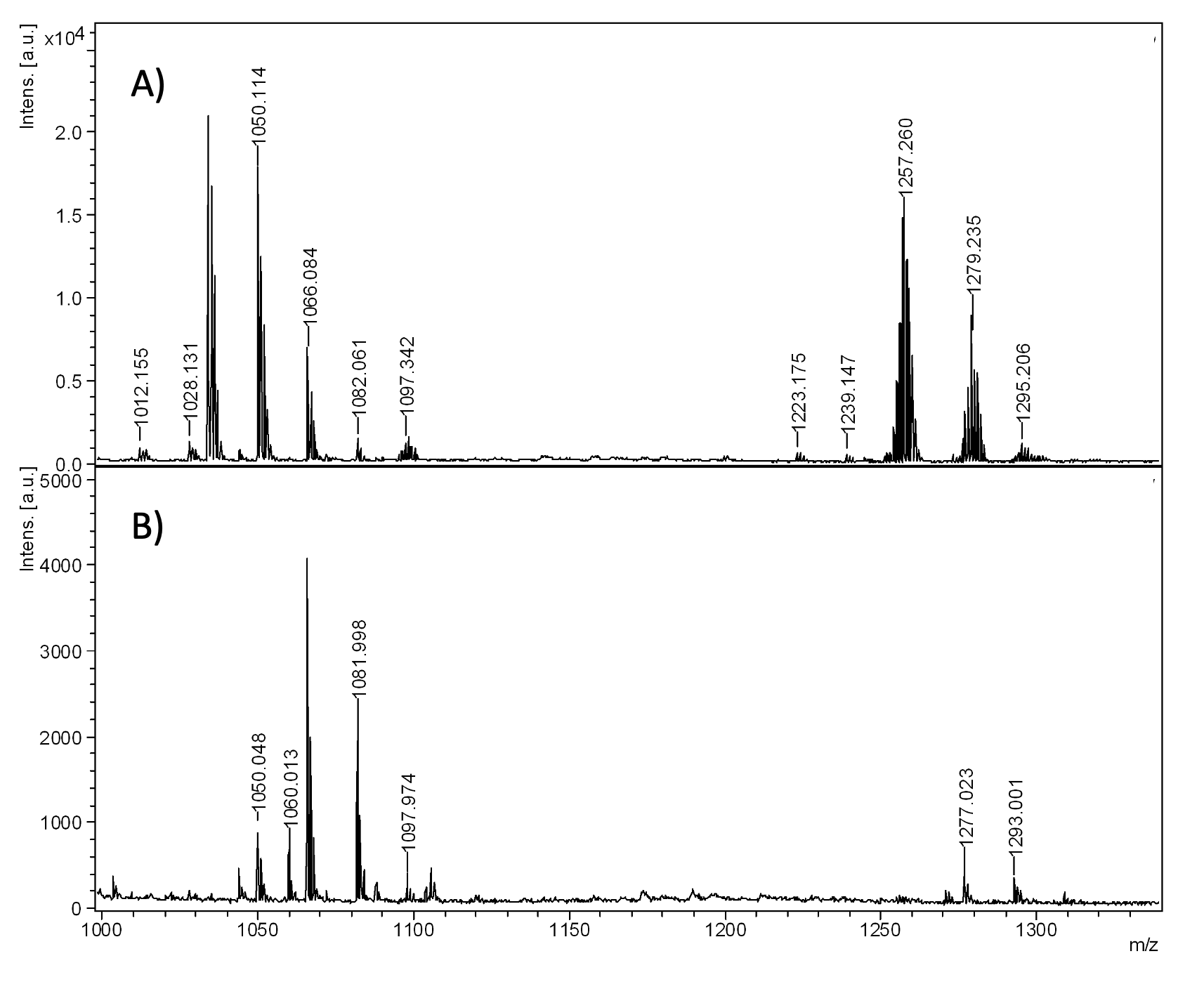

Supplement: Supplementary file 5 [file Image4.TIFF]
